# Anatomy and Physiology

**Published:** 1838-04

**Authors:** 


### PART FOURTH.

### Selections from the British Journals.

(FOR THE QUARTER ENDING FEBRUARY 28, 1838.)

---

### ANATOMY AND PHYSIOLOGY.

*The History of a Female who has four Mammæ and Nipples.* By ROBERT LEE, M.D. F.R.S. Read before the Royal Med. and Chir. Society, Jan. 9, 1838.

THE individual in whom the above-mentioned peculiarity presented itself was thirty-five years of age, and was prematurely delivered of a stillborn child, on the 21st of July, 1835. The mammæ having afterwards become excessively painful and distended, she was compelled most reluctantly to permit the author to make an examination of them; by which it was discovered that she had two mammæ and two nipples on each side. The inferior or pectoral mammæ were fully developed, and in the natural situation; and their nipples, areolæ, and glands presented nothing unusual in their appearance. Near the anterior margin of the axilla, a little higher up on each side, was situated another mamma, about one-sixth the size of the others. The nipples of these were small and flat, but, when gently pressed, a milky fluid flowed copiously and readily from several ducts, which opened upon their extremities. When milk was drawn from the lower breasts, a small quantity usually escaped from the nipples of the upper; and, when the draught came into the former, the latter invariably became hard and distended. From the flatness of the nipples of the upper breasts, the patient had never been able to suckle with them. *Med. Gazette. January* 20, 1838.

---

*Remarks on the Fallacy of* Professor TIEDEMANN'S *Comparison of the Negro Brain and Intellect with those of the European.* By ANDREW COMBE, M.D.

[As we assisted, by a short notice of Tiedemann's paper, (British and Foreign Med. Review, Vol. IV. p. 529,) to disseminate the singular errors which it contains, we deem it the more necessary to notice Dr. Combe's triumphant refutation of them. Dr. Combe's paper, however, like everything that proceeds from the pen of that acute observer and philosophical reasoner, is in itself highly important. If all the advocates of phrenology were possessed of the calmness and philosophical caution displayed by Dr. Combe, in his communications on its principles and phenomena, it would make more rapid progress in the estimation of philosophers and men of science. We take this opportunity of recommending to our readers the journal in which Dr. C.'s paper appears, as containing in every Number much interesting and important information.]

Tiedemann's grand objects are to prove, 1st, that the opinion of Negro inferiority, expressed by Camper, Soemmering, Cuvier, and almost all naturalists of any eminence, is incorrect; 2dly, that the Negro brain is equal in size, and similar in structure, to that of the European; and, 3dly, that, consequently, the former is equally capable of civilization as the latter, and owes his present inferiority entirely to bad treatment and unfavorable circumstances, and will lose it when placed in the position in society which has been recently assigned to him by the "noble British government." These positions are urged by Tiedemann with so much philanthropic warmth, and with such hearty zeal in the cause of the Negro, that we feel no small reluctance to enter the lists against him; but, having a thorough reliance on the supremacy of truth, and believing its diffusion to be fraught with more ultimate happiness to the Negro himself than he can possibly derive from the propagation of an amiable error, we offer no apology for attempting to shew that the prevailing opinion remains unaffected by any evidence brought against it by Tiedemann, and that *de facto* the Negro brain is inferior in intellectual power to that of the European.

In pursuance of the above objects, Tiedemann first enquires whether "the Negro has the same quantity of brain as the European?" and, to ascertain the fact, he institutes an elaborate comparison between the weight of the brain, as determined in upwards of fifty Europeans of different ages and countries, and its weight in several Negroes, examined either by himself or others; and the results obtained are not only full of interest to the phrenologist, but well worthy of the attention of those among our opponents who still continue to ridicule the principle of size of brain being, *cæteris paribus*, a measure of mental power. Every fact mentioned by Tiedemann adds to the already overwhelming proofs adduced by the phrenologists; but, coming in this instance from the pen of a hostile authority, they may probably carry more weight with them than if found in a phrenological essay.

After quoting the statements of many authors, and detailing the weights of fifty-two European brains examined by himself, Tiedemann mentions that "the weight of the brain in an adult male European varies between 3lbs. 2 oz. and 4lbs. 6 oz. troy. *The brain of men who have distinguished themselves by their great talents is often very large:* the brain of the celebrated Cuvier weighed 4lbs. 11 oz. 4 dr. 30 gr. troy, and that of the celebrated surgeon Dupuytren weighed 4 lbs. 10 oz. troy. *The brain of men endowed with but feeble intellectual powers is, on the contrary, often very small, particularly in congenital idiotismus.*" Here, then, is ample confirmation of the phrenological evidence, and from a source which cannot be considered as biassed in our favour. Tiedemann proceeds—"The female brain is lighter than that of the male: it varies between 2 lbs. 8 oz. and 3 lbs. 11 oz. *I never found a female brain that weighed 4 lbs.* The female brain weighs, on an average, from four to eight ounces less than that of the male; *and this difference is already perceptible in a new-born child.* This also corresponds entirely with the long repudiated statements of the phrenologists, and it is pleasant to see the fact thus broadly admitted.

Tiedemann goes even beyond the phrenologists in his applications of the principles of size being a measure of power. He says, "*There is undoubtedly a very close connexion between the absolute size of the brain and the* intellectual *powers and functions of the mind.* This is evident from the remarkable smallness of the brain in cases of congenital idiotismus, few much exceeding in weight the brain of a new-born child. Gall, Spurzheim, Haslam, Esquirol, and others, have already observed this, which is also confirmed by my own researches. The brain of very talented men is remarkable, on the other hand, for its size," (p. 502.) Here certainly is ample corroboration of the influence of organic size on mental power; but Tiedemann has fallen into the very serious error of taking the absolute size of the brain as a measure of intellectual power only; whereas, it indicates, as might be expected *à priori*, absolute *mental* power, without determining whether that power lies in extent of intellect, in strength of moral feeling, or in the force of passion or affection. A brain of four pounds' weight may be large in the anterior lobe and smaller in the middle and posterior lobes; or its chief size and weight may be in the posterior lobes, and the anterior portions be actually small. In both cases, Tiedemann would infer equal "intellectual" power; whereas, the phrenologist would perceive at a glance that, in the former, the intellectual ability would far preponderate, while, in the latter, the power of mind would consist entirely in intensity of feeling, and the intellect, properly so called, be rather weak than strong.

If, for example, we compare the Charib with the Hindoo brain, we find the entire mass of the former considerably to outweigh the latter; and, according to

Tiedemann, we should find more intellectual talent in the Charib. The fact, however, is notoriously the reverse, and the explanation is very easy *when we distinguish the regions of the brain in which the size exists.* In the Charib, the anterior lobe is very small, in perfect harmony with his poverty of intellect; but the posterior and basilar regions of the brain are very large, also in harmony with his ferocity and energy of passion. In the Hindoo, again, the reverse holds: the anterior lobe is well developed, and so is his intellect; but the basilar region, so large in the Charib, is small in him, and, consequently, in vehemence of passion, active courage, and general force of character, the Hindoo is greatly inferior to the more savage Charib.

The same distinction occurs every day in social life. We meet with an individual (a criminal, for instance,) in whom the brain is absolutely large, but who is nevertheless stupid in intellect, and powerful only in the department of the propensities; while, on the contrary, we find many an amiable member of society possessed of a brain smaller in absolute size, but far superior to the criminal in the size of its anterior lobe, or organs of intellect, and consequently far superior to the criminal in thinking power and general talents; results at utter variance with Tiedemann's rule, but perfectly reconcileable according to the phrenological application of the principle.

Hence it is obvious that, of two brains, both precisely equal in absolute weight, one may be very deficient in intellectual endowment compared to the other, and this deficiency be perfectly apparent on inspection, when we attend to the region of the brain in which the preponderance lies. But as Tiedemann, throughout the whole of his experiments, utterly disregards this distinction, confounds intellectual power, moral feeling, and brute propensity under one head, and treats of the brain as if it consisted of only one lobe, with only one function,—namely, the manifestation of intellect,—his inference that, because the Negro brain is equal in weight to the European, therefore the Negro is also his equal in intellectual power, falls to the ground, as unwarranted by the evidence. To render his conclusion worth anything, he must shew, not only that the two brains are equal in absolute size, but that *the anterior lobe, or seat of intellect, is equally developed in both;* a position which he never attempts to substantiate, and which is at variance with some parts even of his own facts.

Having obtained the weight of a sufficient number of European brains, Tiedemann next endeavours to ascertain the weight of the Negro brain; but, from the very small quantity of Negroes to be found in Europe, he has great difficulty in obtaining anything like a fair average: in fact, he gives the weight of *only four* Negro brains: one of a boy of fourteen years of age, stated, on the authority of Soemmering, to have weighed 3 ℔s. 6 oz. 6 dr.; a second, of a tall and handsome Negro, of twenty years of age, which weighed 3 ℔s. 9 oz. 4 dr.; a third, of a large Negro, mentioned by Sir Astley Cooper, of 49 oz.; and a fourth, examined by himself, of a man twenty-five years of age, which weighed 2 ℔s. 3 oz. 2 dr.

In comparing these results with the average weight of the European brain, as stated by Tiedemann himself, it is singular to observe the extent to which they are at variance with his inferences. The European average runs, he says, from 3 ℔s. 2 oz. to 4 ℔s. 6 oz., while the average of the four Negroes rises to only 3 ℔s. 5 oz. 1 dr., or 3 oz. above the *lowest* European averages; and the *highest* Negro falls 5 oz. short of the highest *average* European, and no less than 10 oz. short of Cuvier's brain. And, as if these facts were not inconsistent enough with his conclusions, Tiedemann first affirms that in the Negro "the length and height of the cerebral hemispheres do not visibly differ from that of the European, their breadth only being somewhat less," (p. 515;) and immediately after subjoins three tables of the "Dimensions of the Cerebrum of Negroes," "Dimensions of the Cerebrum of European Males," and "Dimensions of the Cerebrum of European Females;" the figures of which directly contradict his assertion! This seems almost incredible; but, on summing up the averages, we find the following results, namely,

| | | | Inches. | Lines |
|---|---|---|---|---|
| Average length of brain in | 4 Negroes | . | 5 | 11 |
| | 7 European males | . | 6 | $2\frac{1}{7}$ |
| | 6 females | . | 5 | $10\frac{1}{2}$ |
| Average greatest breadth in | 4 Negroes | . | 4 | $8\frac{1}{6}$ |
| | 7 European males | . | 5 | $1\frac{1}{7}$ |
| | 3 females | . | 5 | $4\frac{1}{3}$ |
| Average height of brain in | 3 Negroes | . | 2 | $11\frac{1}{3}$ |
| | 7 European males | . | 3 | 4 |
| | 4 females | . | 2 | $9\frac{1}{2}$ |

From these tables it is evident that the dimensions of the brain are smaller in the Negroes measured by Tiedemann than in the European; but, for our own parts, we are not disposed to lay much stress upon results drawn from such a limited number of facts, and we notice them merely to shew that, such as they are, they directly contradict the arithmetical proportions or conclusions drawn from them by Tiedemann. The latter, indeed, grants all that we contend for, when, in his description of the Negro brain, (p. 515,) he states that "*the anterior portion of the hemispheres is something narrower than is usually the case in Europeans*," because, as the anterior portion is the seat of *intellect*, this is really equivalent to conceding that the Negro *is* naturally inferior in intellectual capacity to the European.

Not having access to a sufficient number of the actual brains of Negroes, Tiedemann has endeavoured to supply the want of direct evidence by comparing the capacity of the Negro skull with that of the European, and thus obtaining an index to the relative size of their contained organs. For this purpose he filled the skulls with millet-seed, and carefully noted the quantity which each contained."

. . . .

After giving several pages of tables comprehending the weight of the quantity of millet-seed required to fill Ethiopian, Caucasian, Mongolian, American, and Malayan skulls, Tiedemann says, "It is evident, from the comparison of the *cavum cranii* of the Negro with that of the European, Mongolian, American, and Malayan, that the cavity of the skull of the Negro, in general, *is not smaller* than that of the European and other human races. The result of Hamilton's researches is the same. I hope this will convince others that the opinion of many naturalists, such as Camper, Soemmering, Cuvier, Lawrence, and Virey, that the Negro has a smaller skull and brain than the European, is *ill-founded, and entirely refuted by my researches*." (P. 511.) Now, we have already seen that the real question of interest, as regards Negro improvement, is not so much the general size of his brain as the relative size of its anterior lobe and coronal surface compared to the basilar and posterior portions: but, even as concerns the absolute size of the whole brain, it is an extraordinary fact that Tiedemann's own tables give a decided superiority to the European over the Negro, to the average extent of nearly four ounces! The average capacity of forty-one Negro skulls in his own tables amounts only to 37 oz. 1 dr. 10 gr., while the average of seventy-seven Europeans of every nation, also in his own tables, amounts to 41 oz. 2 dr. 30 gr. Of the Negroes, indeed, three are females; but, even subtracting these, the Negro average amounts only to 37 oz. 6 dr. 18 gr. Here, then, on Tiedemann's own shewing, we have, first, an inferiority in the dimensions of the Negro brain and a greater narrowness of its anterior lobe; and, secondly, a marked inferiority in the capacity of the Negro skull to the extent of about one-tenth, and yet he very strangely infers that *both are equal* to those of the European. . . .

That a physiologist of Tiedemann's talent and merited reputation should have failed so signally in an investigation which he recognizes as one of so much importance, and upon which he has bestowed so much labour, and with so benevolent an intention, is much to be lamented; but the cause which has led to his failure is still more to be lamented, because it is humiliating to him as a man of science, and is the natural and just result of his own conduct. Well did Tiedemann know that the great discovery of his immortal countryman, Gall, lay directly in his way in the enquiry in which he was engaged; and that, if true, it must be of immense use to him in conducting his enquiry. Had he availed himself of its aid, he would have seen at once the futility of any investigation based on considering the whole brain as the organ of *intellect*, and would thus have avoided becoming the instrument of authoritatively diffusing mischievous error, where he was anxious only for beneficent truth. Tiedemann, however, confiding in the strength of his own merits and the durability of his own fame, chose to treat the phrenological physiology of the brain with contemptuous silence, to disregard its facts, and to reject its aid as a guide. He has preferred being a leader in the train of error to being a subordinate in the march of truth; and, as he has chosen his path, so shall he be rewarded. His contribution to the Royal Society's Transactions, although hailed at present as an honour to its author, will ere long be regarded as a beacon to warn others how very little a first-rate talent, great industry, and a European reputation can accomplish when employed in a false direction, and how indispensable to true greatness is the direct and undeviating pursuit of truth.

*Phrenological Journal.* No. liv. *Dec.* 1837.

---

*Second Report of the London Committee of the British Association for the Advancement of Science, on the Motions and Sounds of the Heart.* Communicated at the Meeting at Liverpool, September 1837.

The Committee appointed in London to investigate the motions and sounds of the heart, have to present to the Association a short account of some investigations of the abnormal sounds of the heart and arteries, in which they have been recently occupied, and which were not comprehended in their former Report.

Before describing these, the Committee would remark, that although their enquiries have not been specially directed to that subject, yet they have had many opportunities of verifying the conclusions on the natural sounds, as presented in their Report of last year; and these conclusions not having been since shaken by any experiment or rational objection, it may be considered as fairly established, that the first, or systolic sound of the heart, is essentially caused by the sudden and forcible tightening of the muscular fibres of the ventricles when they contract; and that the second sound, which accompanies the diastole of the ventricles, depends solely on the reaction of the arterial columns of blood on the semilunar valves at the arterial orifices. It further appears, that the first sound may be increased by an additional sound of impulsion against the walls of the chest under certain circumstances of posture, of increased action of the heart, and of particular stages of the respiratory act. It is also obvious, that the character of the first sound may in some measure depend on the closure of the auriculo-ventricular valves, and on the quantity of blood, inasmuch as these determine the nature and time of the resistance, against which the muscular fibres of the ventricles tighten. So likewise the vigour of the ventricular systole, the quantity of blood propelled by it, the sudden and complete character of the diastole, the fulness of the arterial trunks, as well as the perfect, mobile, and membranous condition of the semilunar valves, will determine the character and loudness of the second sound. An experimental illustration of one of these conditions was observed by the Committee during one of their experiments on the ass—a great diminution of the second sound on the carotid artery being freely divided.

As additional illustrations of the production of a sound like that of the heart, by muscular contraction, the Committee have noticed that accompanying the action of the panniculus carnosus of the ass during life, and the quivering contraction of muscles immediately after death. The sound produced in the latter case, in nature and frequency, closely resembled the first sound of the heart of the fœtus or of small animals.

In investigating the morbid sounds of the heart, the attention of the Committee has been chiefly directed to the causes of those remarkable and various accompaniments of the heart's action, called murmurs, which were happily compared by Laennec with the noises of blowing, filing, rasping, sawing, purring, cooing, &c.

This enquiry consists of two parts. 1. What is the essential physical cause of the phenomena in question? and 2, How does the apparatus of the circulation develop this cause in the various instances in which these phenomena are known to occur? To the first of these enquiries, the experiments of the Committee supply what they trust will be deemed a satisfactory answer; the second is to be answered by extensive clinical and pathological observation, rather than by direct experiment; and although a few physiological experiments will be quoted to this point, yet the Committee do not profess at present to do more than open this enquiry to all those who have the means of pursuing it.

*Experiments on the production of Sound by the motion of Water through a Tube.*

1. A caoutchouc tube, eighteen inches long, and three-eighths of an inch in diameter, was attached to the stop-cock of a reservoir, in which there was water to the depth of from eight to ten inches.

When the water flowed perpendicularly through this tube (the air being first expelled,* and the lower end of the tube kept under water in the recipient below,) no sound was heard; but on pressing any part of the tube, so as to diminish its caliber, a blowing sound was heard at and below the point of pressure, and this sound became louder and more whizzing as the pressure was increased. The loudest sounds were obtained at the lowest end of the tube, when they were sometimes quite musical; and by increasing the pressure at regular intervals, a periodic increase and raising of the sound was produced, which closely resembled the sound heard in the neck, to which the French have given the name of "bruit-de-diable."

2. A pin being stuck transversely through the tube, a slight blowing was heard. A similar phenomenon more distinctly resulted from the use of a split goose-quill placed in the same way. A stronger blowing was produced by two threads across the diameter of the tube, especially when they were rather loose; and a still louder and shriller sound ensued when a knot of string was fastened to the threads.

3. The same tube being adapted to the stopcock of a water supply-pipe, through which the current could be let to pass with great force, it was found possible to imitate every variety of blowing, whizzing, and musical murmurs, by varying the pressure on, or obstruction in, the tube, and by altering the force of the current. When the current was strong, the least obstruction caused a murmur, but, with weaker currents, greater obstructions became necessary for the same effect. An obstruction which, with a weak current, gave a blowing sound, produced with a stronger current a sound of a more whizzing character. Grating or rasping sounds were best obtained by the effect of a strong current on a knotted thread across the diameter of the tube. The musical or uniform sounds resulted from a moderately strong current through a considerable impediment; increasing the force of the current or the degree of obstruction, rendered them whizzing and imperfect; diminishing the current or the obstruction, converted them into a simple blowing. When a sound was of an appreciable pitch, its note was high in proportion to the force of the current and the amount of the obstruction, a fine forcible stream producing the highest note. Sometimes, however, with a strong current, a loud trumpet-note would be set up, which was not altered in pitch, but only in force, by changing the strength of the current. This kind of note produced visible vibrations of the tube below the obstruction, and seemed to have relation to the length of the tube. In many instances these vibrations resembled closely the purring tremor and thrilling vibration sometimes felt in the region of the heart and large arteries. Musical sounds of a more variable character, like the cooing of a dove, the humming of an insect, or the whistling of wind, were produced with weak currents passing through a tube much obstructed. The pressure of a column of water only two or three inches high was sufficient to give acute whistling notes, which were sustained, although varying, even when the water that passed only fell in drops from the end of the tube.

4. Bending the tube to an angle produced a murmur; but no sound resulted from any curve that did not infringe on the caliber of the tube. A circular constriction, by a thread drawn round the tube, caused a murmur, which was blowing or whizzing according to the strength of the current.

5. The current issuing from the end of a tube, or from the mouth of an India-rubber bottle, produced a blowing sound when it impinged directly on an opposite surface, such as the side of the recipient or the end of the stethoscope, but unless the current were very strong, this sound was not produced when the current played on the surface very obliquely.

6. When the tube, with a weak current, was pressed on at two points, the murmur was heard at the point where the pressure was greatest; and by increasing the pressure at one point, the sound was stopped at the other. When the current was strong, it was easy, by a pretty equal pressure, to cause a murmur at both points.

7. With a strong caoutchouc tube, two feet long, and one inch in internal diameter, the same results were observed, but in a more remarkable degree, in consequence of the increased size of the tube. When the current was strong, and the pressure on the tube considerable, sounds were produced loud enough to be heard without applying the stethoscope to the ear, and the vibrations of the tube below the obstructions were so strong that they threw the water in little jets from the outside of the tube.

8. In making the last experiment, the pressure of the water suddenly distended a portion of the tube into a globe about three inches in diameter, constituting a good representation of a circumscribed true aneurism. As long as the force of the current was sufficient to keep the walls of the dilated portion distended, no murmur was produced; but when these walls became flaccid, the passing current caused a kind of dull fremitus in them. Slight pressure on the dilatation or bending the tube to form an angle at the point, also sometimes occasioned a murmur.

9. A globular India-rubber bottle, three inches in diameter, being adapted to an aperture in the side of a tube, so as to form an elastic sac communicating with it, and the air being expelled, the current of water was directed through the tube. The same was done with a smaller tube, and a bottle one inch and a half in diameter. In some positions the current, in passing the lateral sac, occasioned a slight whizzing; but in others, as when the tube was straight, there was no sound. A sudden increase of current, or the removal of pressure from the sac, caused a whizzing, by the entry of water into the sac. Independently of the current, sudden forcible pressure on the sac occasioned a whizzing with the expulsion of the fluid; and a similar whizzing attended its rapid reflux into the sac on the removal of the pressure.

10. Some of the preceding experiments being repeated with water made slightly glutinous with size, it was found that the various sounds were not quite so readily produced as with plain water, and required a greater force of current.

From all these results (1, 2, 3, 4, 5, 6, &c.) it is sufficiently plain that a certain resistance or impediment to a liquid current is the essential physical cause of all murmurs produced by the motion of fluids in elastic tubes. That any condition of the walls of the tube beyond the obstructing point is not, as it has been supposed, essential to the production of these sounds, is proved by the fact, that they may be produced by an obstruction at the terminal orifice of the tube, or at the mouth of a gum-elastic bottle, where there is no tube or wall beyond to cause them (1, 5); and usually this is the situation where they can be produced best, because here the current has acquired its greatest momentum, and finds a freer passage beyond the obstructed point. The more flaccid state of the portion of a tube beyond an obstruction is a necessary effect of the impediment caused by that obstruction to the passage of water. It is therefore a necessary concomitant of the obstruction, but it is not the cause of the sound. When, however, the sound occasioned by the obstruction is strong, its vibrations may be communicated to the whole contents and walls of the tube beyond (3, 7,) which will then vibrate in system with it, and be capable of modifying its note; just as the tube of a reed instrument affects the note which is generated exclusively in the reed. On the other hand, when the sound is weak and varying, the condition of the tube or walls beyond it will not affect it. In short, the laws of the production of sound by liquids so closely resemble those which regulate the same phenomena in air, that it is unnecessary to enter into further detail respecting them. It may be necessary to advert to an objection to this view, that a murmur is sometimes caused where there is no impediment to the course of a liquid, as when it passes suddenly from a small into a large tube or into a sac. In the first place, it is not quite correct to assume that in this case there is no impediment; for the liquid in the large tube or sac, having less velocity than that in the small one, must itself be an impediment. But besides this, the course of the small swift current becomes changed by spreading into the larger channel, and instead of running smoothly parallel to the tube, now strikes its walls at an angle, causing a series of impulses and resistances which, if forcible and rapid enough, constitute the vibrations of sound. It is in the same way that a current produces a sound by impinging against an opposed surface (5.) It may be observed, however, that these indirect kinds of impediment to a moving current, are not so constantly attended with the production of sound, as the direct obstacle presented by a narrowing of, or projection into, the caliber of the tube.

*Experiments on the production of Murmurs in the Living Body.*

11. About two inches length of the common carotid artery of a young donkey was laid bare. Different degrees of pressure, either by the stethoscope, or by a probe pushed under the artery, occasioned a variety of murmurs, blowing, sawing, filing, and musical or cooing, at each pulse. When the stethoscope was merely placed in contact without pressure, no murmur was heard, but only a simple impulse and sound, which was distinct only when the heart acted strongly.

12. The artery was scratched for a few seconds with the point of a scalpel; it gradually became sensibly smaller for the length of half an inch about that point. A strong solution of salt being applied, the contraction increased; but it was still of a gradual and tapering kind, and the stethoscope could not detect any murmur in it; very slight pressure was enough to cause a whizzing. The pulse at this contracted portion was felt to be much harder and sharper than above or below it.

13. A small incision being made into the artery, a jet of blood issued, and a whizzing, sometimes continuous like the bruit-de-diable, sometimes only in pulses, was heard beyond the orifice, but no sound on that side of the orifice nearest the heart; the sound being, as usual, carried in the direction of the current. The incision being made larger, the blood spouted out with whizzing to the distance of more than six feet, and the animal died in the course of ten minutes. After this last incision the beats of the heart were frequent, short, and pretty loud, but without a second sound, and without any murmur to the last. They continued for nearly two minutes after the respiration and consciousness had ceased, becoming gradually slower.

14. The Committee repeated the observation that has often been made before, that a murmur can easily be produced by pressure on the subclavian, carotid, or femoral artery of the human subject. This murmur is generally of a grating or filing character, and is prolonged in proportion to the degree of pressure.

15. Whilst making the observations on the carotid, they found that a continuous murmur of very remarkable and variable characters could be produced by pressure on the jugular veins. The most common sound thus produced was like the humming of a gnat or fly, but occasionally it resembled the whistling of wind, the singing of a kettle, the cooing of a dove, and sometimes it was perfectly the remittent whirring noise, which the French have called the bruit-de-diable. Dr. Ogier Ward, of Birmingham, had previously come to the conclusion that this sound is produced in the jugular veins; and the observations of the Committee confirm this inference; but they do not agree with this physician in the opinion which he adopts from MM. Andral and Bouillaud, that the presence of these sounds always denotes a chlorotic state of the system, in which steel is indicated, or that they are essentially morbid symptoms at all. They may be produced in the healthiest subjects by moderate pressure applied to the lower part of the jugular veins, and are then found to be modified by various circumstances, which can only affect the venous current. Thus, they may be arrested or diminished by pressure on the vein above, by the horizontal posture, or hanging down the head, and by forced efforts to expire with the glottis closed. They may be restored in increased degree by suddenly desisting from any of these acts or circumstances. The occasional pulsatory or remittent character of these sounds seems to depend on the momentary increase of pressure caused by each pulse of the neighbouring artery; and when, as it oftentimes happens, these pulses are attended with a whizzing, this is in a measure incorporated with the venous sound, and increases its periodic swell. The size and downward current of the jugular vein peculiarly adapt it for the production of sound, but it is probable that sounds may be produced by pressure on other veins, when circumstances accelerate the current within them. The Committee have succeeded in detecting an obscure murmur in some of the large superficial veins in the arm and thigh. It is to be distinguished from muscular sound, which it resembles, by its being heard only when the small end of the stethoscope is applied on a vein, and by its being stopped by pressure on that vein. A louder continuous sound is sometimes heard at each side of the upper part of the sternum, which, from its resemblance to these venous sounds, and from its being stopped by forcible expiration, may be supposed to have its seat in the large venous trunks underneath.

Although it appears from these facts that the venous sounds are not necessarily signs of disease, yet the circumstance proved by the Committee (10), that water is thrown into sonorous vibrations more readily than a liquid of a more glutinous chatacter, renders it probable that these, and other sounds depending on the motion of liquids in the apparatus of the circulation, may be more easily produced where the blood is thin and deficient in quantity; and under these circumstances they may occur in the neck from the mere pressure of the muscles on the jugular veins.

The Committee had planned several experiments for the further elucidation of the second part of the enquiry, namely, by what changes, functional and structural, does the apparatus of the circulation develop the physical causes of abnormal murmurs and sounds in various instances in which they are known to occur? Having failed in obtaining animals in time for this Report, the Committee propose to resume at a future time this part of the enquiry, so important for the elucidation of several obscure points in pathology, diagnosis, and practice; and to report the result of their labours at the next meeting, if the Association should think fit to reappoint them for that purpose.* (Signed)

C. J. B. WILLIAMS, M.D. F.R.S.,
ROBERT B. TODD, M.D., Professor of Physiology, and General and Morbid Anatomy, King's College, London.

*Med. Gazette. December* 2, 1837.

---

*On the Cerebral Extremity of the Optic Nerve.* By SAMUEL SOLLY, Esq.

I AM desirous of putting upon record a fact regarding the cerebral extremity of the optic nerve, which I do not find noticed in any author on this subject. It may be interesting to many, inasmuch as it appears to afford additional evidence of the important office of the cineritious neurine; especially when taken in connexion with those views of the function of the grey matter which Mr. Grainger has lately so materially elucidated by his minute dissections of the central extremities of the spinal nerves, and the deductions which he makes in confirmation of Dr. Marshall Hall's views of the spinal cord as a true centre of power. I will also take this opportunity of bearing my testimony to the accuracy of Mr. Grainger's statement regarding the roots of the spinal nerves.

I traced fasciculi of both the anterior and posterior roots of the spinal nerves, into the grey matter of the cord, in presence of Dr. Todd, at King's College, on a dog which he had procured for that purpose; since which, I have seen them exposed by Mr. Grainger himself, at his school in Webb-street.

The connexion of the optic nerve with the brain itself has been variously described by different authors; by some it has been described as arising only from

* The Committee was reappointed, and 25*l.* placed at their disposal for the further prosecution of these researches.

the thalami nervorum opticorum; by others, only from the optic tubercles; and by most, in latter days, from both of these bodies, "by a flat band of white fibres." These fibres, which come from the surface of the thalamus and the optic tubercles, are well known and easily demonstrated; but they are not the only fibres of communication between this nerve and the brain.

If the optic nerve is carefully traced from its commissure backwards, it will be found to be connected to the tuber cinereum, as described elsewhere; after crossing the crus cerebri, to which it is connected with membrane, it divides into a superficial and deep layer: the superficial layer is that which is described in most anatomical works; the deep layer, which is thin and flat, plunges partly into the substance of the thalamus and partly into the corpus geniculatum. Those fibres which go through the corpus geniculatum are separated into delicate threads by cineritious neurine, as the motor tract of the spinal cord in the corpus striatum. These fibres which plunge into the thalamus are stronger and more distinct, and after spreading into rays are lost in its substance.

This arrangement may be seen after raising the nerve from the crus cerebri, either by tearing its fibres very carefully in a brain previously hardened in alcohol, or by making a longitudinal perpendicular section of the optic nerve in a recent brain, right through the corpus geniculatum and thalamus; when one layer of white neurine will be seen on the surface of the corpus geniculatum, and another just passing through its anterior part, but principally through the substance of the thalamus, separated from the first by the posterior and superior portion of the grey matter of the corpus geniculatum.

*Med. Gazette. February* 24, 1838.

